# Toxic epidermal necrolysis after the administration of enfortumab vedotin for urinary bladder urothelial carcinoma

**DOI:** 10.1002/iju5.12562

**Published:** 2022-12-02

**Authors:** Yuji Mimura, Aya Kobayashi, Haruhiko Utazu, Yuki Matsumoto, Hiroya Mizusawa

**Affiliations:** ^1^ Department of Urology, NHO Shinshu Ueda Medical Center Ueda Nagano Japan; ^2^ Department of Dermatology Shinshu Ueda Medical Center Ueda Nagano Japan

**Keywords:** adverse event, antibody‐drug conjugate, cutaneous toxicity, metastatic bladder cancer, Stevens–Johnson syndrome

## Abstract

**Introduction:**

Enfortumab vedotin is a novel drug for locally advanced or metastatic urothelial carcinoma, but it is associated with a high incidence of skin reactions (up to 47.0%).

**Case presentation:**

A 71‐year‐old male was administered enfortumab vedotin for bladder cancer associated with lymph node metastases. Slight erythema of the upper limbs appeared on Day 5. Erythema gradually worsened. On Day 8, second administration was performed. On Day 12, based on the extents of blisters, erosion, and epidermolysis, a diagnosis of toxic epidermal necrolysis was made. The patient died of multiple organ failure on Day 18.

**Conclusion:**

As serious cutaneous toxicity may appear early after the start of administration, it is important to consider the timing of the second administration of the initial course carefully. In cases of skin reaction, reduction or discontinuation should be considered.

**Abbreviations & Acronyms**
CT: computed tomography
EV: enfortumab vedotin
SJS: Stevens–Johnson syndrome
TEN: toxic epidermal necrolysis

**Keynote message**

As serious cutaneous toxicity may appear early after the start of enfortumab vedotin administration, urologists/oncologists need to consider the timing of the second administration carefully, especially in the initial course. In cases of cutaneous toxicity, reduction or discontinuation should be considered without hesitation.

## Introduction

EV is an antibody‐drug conjugate for non‐resectable urothelial carcinoma. In Japan, this drug was approved in 2021. We experienced a patient who developed TEN after EV administration. We report this adverse event, and review case reports in other countries where EV had been clinically used prior to approval in Japan.

## Case presentation

**Fig. 1:**
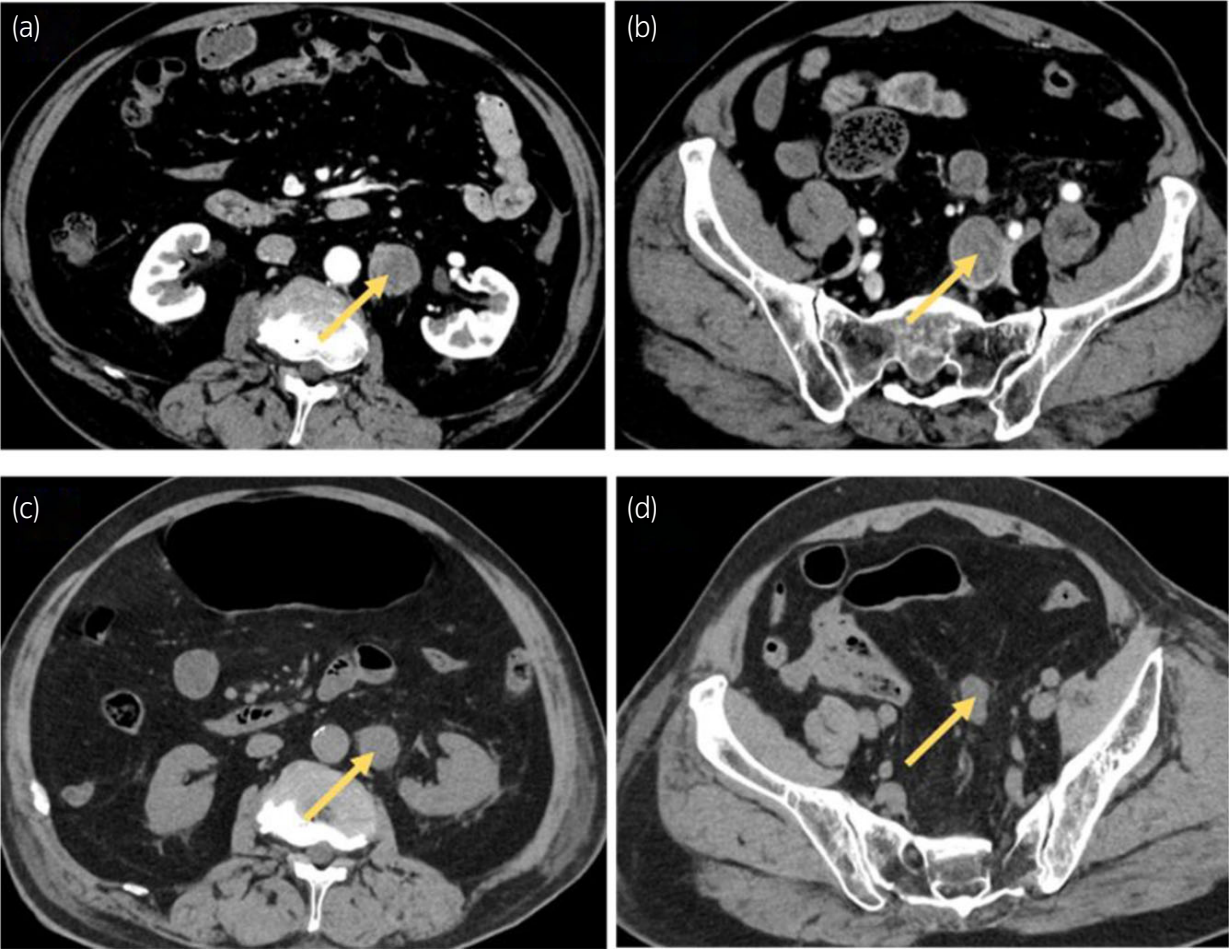
Abdominal CT. (a, b) Contrast‐enhanced CT retroperitoneal and intrapelvic lymph node metastases before EV administration (arrows). (c, d) Plain CT lymph node metastases (arrows) are slightly reduced 16 days after the start of EV administration.

A 71‐year‐old male. Concomitant diseases, hypertension, alcoholic hepatitis, hyperuricemia, and asteatotic dermatitis were present. He had no drug allergies. He received drug therapy. He had undergone transurethral resection five times for bladder cancer. In the fifth session, pathological findings suggested invasive urothelial carcinoma, G3, pT1. Considering radical cystectomy, two courses of chemotherapy (4 weeks schedule; gemcitabine 1000 mg/m^2^ Day 1, 8, 15, cisplatin 70 mg/m^2^ Day 2) were performed. Intrapelvic lymph node metastasis was detected. Subsequently, 10 courses of immune checkpoint inhibitor therapy (pembrolizumab 200 mg/body every 3 weeks) and two courses of chemotherapy (gemcitabine 1000 mg/m^2^ Day 1, 8, 15; paclitaxel 180 mg/m^2^ Day 1) were performed. Lymph node metastasis was exacerbated (Fig. 1), and he was admitted for EV administration.

On admission, 11 weeks after final chemotherapy, height and body weight were 162.1 cm and 77.7 kg, respectively. Consciousness was clear. Blood pressure, pulse, body temperature, and oxygen saturation were 142/88 mmHg, 88 times/min, 36.0°C, and 97% (indoor), respectively.

**Fig. 2:**
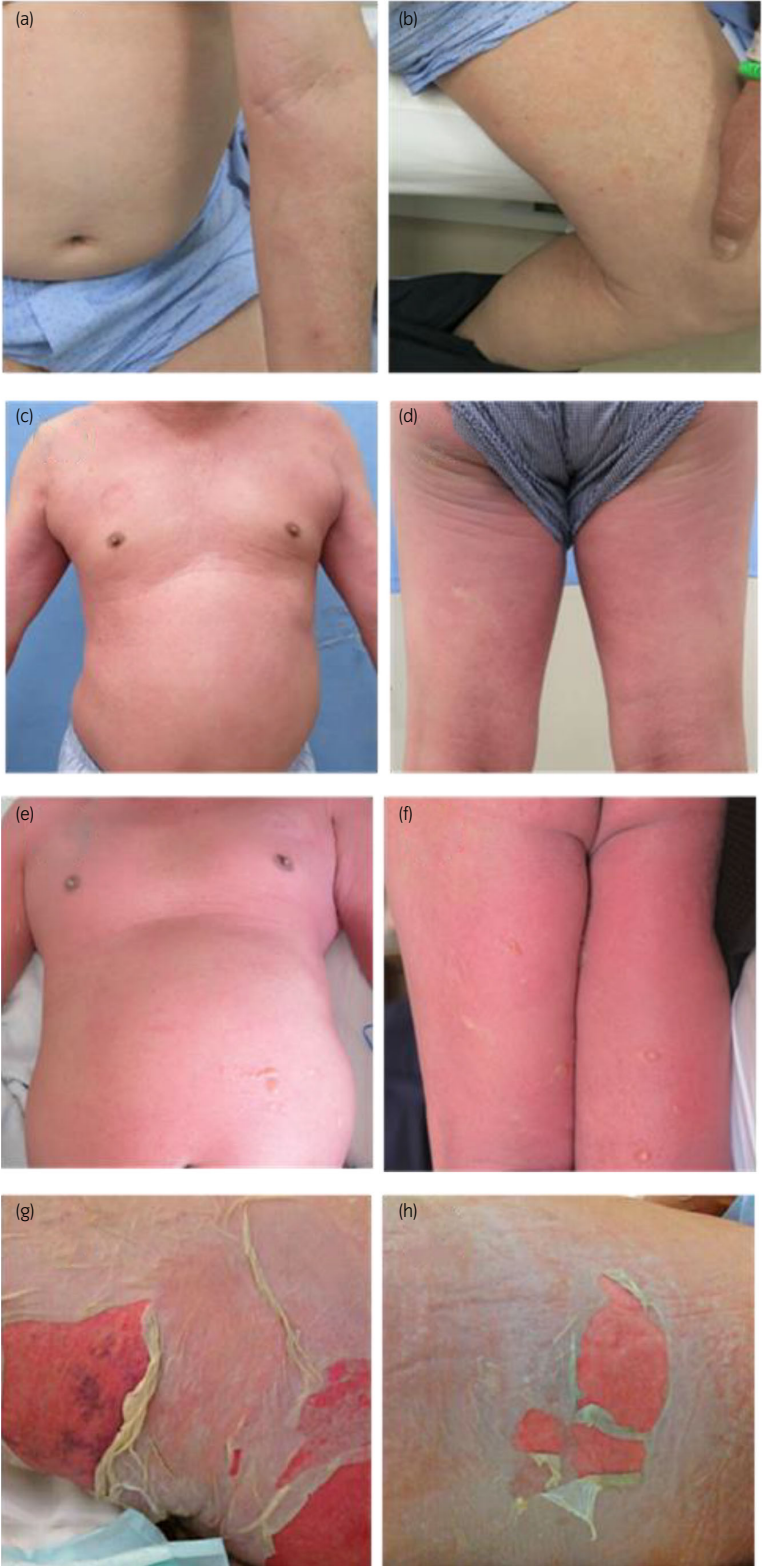
Macroscopic findings of the skin. (a, b) Day 6 of administration. Slight edematous erythema of the upper limbs and slight erythema of the medial thighs are observed. (c, d) Day 10 of administration. Extensive erythema of the upper limbs/trunk/thighs is noted. (e, f) Day 12 of administration. Extent of erythema further increased, and blister formation is observed. (g, h) Day 15 of administration. Erosion and epidermolysis are exacerbated.

**Fig. 3:**
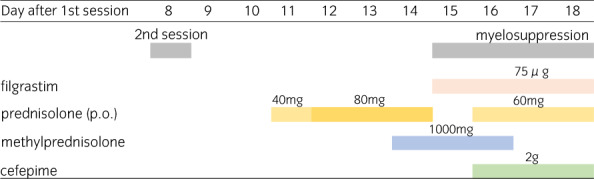
Progress chart of administration of steroid and antibiotics.

Very slight erythema of the upper limbs with pruritus appeared 5 days after the start of EV (1.25 mg/kg) administration. Slight edematous erythema of the bilateral upper limbs as well as slight erythema of the entire abdomen and bilateral medial thighs were noted on Day 6 (Fig. 2), and he consulted the Department of Dermatology. Management of levocetirizine hydrochloride and 0.05% betamethasone butyrate propionate ointment was continued. On Day 7, both erythema and pruritus were further exacerbated. On Day 8, his general condition was fair except for erythema and pruritus, and a second session of EV administration was performed after consulting a dermatologist (Table 1). On Day 10, slight fever, fatigue, and erythema of the upper limbs/trunk/thighs were observed (Fig. 2). On Day 11, blister formation was noted in a portion of erythema, and the administration of prednisolone was started (Fig. 3). On Day 12, systemic erythema and blister formation were further exacerbated. There was no enanthema. Some blisters ruptured, leading to erosion formation (Fig. 2). Skin biopsy was performed. Liquefaction degeneration at the dermo‐epidermal junction was marked, and subepidermal blister formation was observed. Based on the extent of blisters, erosion, and epidermolysis, a diagnosis of TEN was made. On Day 15, erosion formation was observed, epidermolysis further deteriorated, and a large amount of exudate was observed (Fig. 2). Hematology showed marked leukopenia and deterioration of renal function (Table 1). The third session of EV administration was discontinued. Thoracoabdominal computed tomography revealed that lymph node metastasis was slightly reduced (Fig. 1). On Day 16, the patient was referred to the High Care Unit for ventilator management, and hemodialysis for acidosis was started. On Day 18, the patient died of multiple organ failure.

**Table 1:** Changes in hematological data before and after EV administration

	Before administration	Day 8 of administration	Day 13	Day 16	Day 18
Alb, g/dL	3.6	2.9	2.9	2.4	2.4
AST, U/L	49	38	42	21	90
ALT, U/L	30	32	34	35	64
BUN, mg/dL	16.7	9.9	16.3	26.8	25.5
Cr, mg/dL	0.68	0.66	0.72	1.97	2.32
Na, mEq/L	139	138	132	130	138
K, mEq/L	4.4	3.8	4.1	4.6	4.2
Cl, mEq/L	107	106	99	97	101
CRP, mg/dL	0.7	1.1	3.9	16.9	30.9
WBC, ×10^2^/μL	54	39	34	6	9
Neut., %	54.4	53.4	78.4	28.6	46.1
Eos., %	2.2	3.6	0	0	0
RBC, ×10^4^/μL	438	408	359	379	332
Plat, ×10^4^/μL	10.6	9.6	9.9	4.7	1.4

Alb, albumin; ALT, alanine transaminase; AST, aspartate transaminase; BUN, blood urea nitrogen; Cl, chlorine; Cr, creatinine; CRP, C‐reactive protein; Eos., eosinophil granulocyte; K, potassium; Na, sodium; Neut., neutrophil, Plat, platelet; RBC, red blood cell; WBC, white blood cell.

## Discussion

Stevens–Johnson syndrome (SJS) and TEN are known as severe representative drug eruptions characterized by epidermal necrosis and mucosal erosion. TEN refers to severe SJS in which the blister/erosion area accounts for ≥30% of the body surface area. Risk factors include an advanced age and underlying diseases, including malignant tumors. The prognosis of patients with TEN is poor, and the mortality rate is reportedly ≥40%.^1^, ^2^

It is indicated that EV may more frequently induce skin reactions due to the dermal expression of its target, nectin‐4. The incidence of skin rashes related to antibody‐drug complexes with a similar mechanism is high.^3^ In a previous phase III clinical trial, EV was administered to 296 patients, and incidences of skin reactions were 47.0%. Mostly they appeared in the first course, and the median period was 0.46 months after EV administration.^4^ There was no patient with SJS/TEN; however, other clinical trials reported SJS/TEN.^5^ In clinical trials, patients with concomitant diseases or those with a poor general condition were excluded; thus, attention must be paid in real clinical practice to such patients. Especially, the package insert of EV warns about adverse events in patients with liver dysfunction.

In the United States, EV had been used in clinical practice prior to its approval in Japan. Nguyen *et al*.^6^ examined the development of SJS/TEN in the United States during a 10‐month period after EV approval based on post‐marketing survey reports and information from PubMed. The subjects were eight patients definitively diagnosed by dermatologists or through pathological diagnosis. The median interval from EV administration until the onset of SJS/TEN was 11 days. The frequency of EV administration was twice (*n* = 6) and three times (*n* = 1). In all patients, the onset of SJS/TEN led to serious outcomes. Four patients died of SJS/TEN, and the other four patients required treatment in the intensive care unit or burn care unit. The detailed courses of the patients who died were also reported.^7^, ^8^

According to the fifth post‐marketing survey/interim report on adverse reactions in Japan (during a 6‐month period after approval), SJS and TEN were observed in seven patients each.

There were case reports of SJS/TEN that was related to the administration of antitumor drugs to treat urothelial carcinoma. Maloney *et al*.^9^ collected data from 18 patients with SJS/TEN related to immune checkpoint inhibitors. The median intervals from the administration of nivolumab or pembrolizumab until the onset of SJS/TEN were 3 and 11 weeks, respectively. No patient died of SJS, but three of five patients with TEN died. Thus, it is possible that a drug other than EV‐induced TEN.

The frequency of EV‐induced SJS/TEN may be higher than that of SJS/TEN for conventional antitumor drugs. When reviewing case reports, including the present case, the interval from administration until onset is very short,^5^, ^6^ and ocular/dermal symptoms must be sufficiently checked in the initial phase of administration. Thus, cooperation with a dermatologist is recommended before the first session.

It is difficult to predict the development of SJS/TEN. On Day 8, we judged that erythema was an adverse event (grade 2); therefore, we administered EV and consulted a dermatologist. When a cutaneous adverse event is grade 3 or higher, instructions for reduction of the administration dosage or discontinuation are shown on the official package insert. However, reduction/discontinuation should be considered even if they are grade 1 or 2. Moreover, myelosuppression may appear after the second session; thus, the patient's general condition is expected to worsen. In the present case, the period of serious cutaneous toxicity overlapped the period of myelosuppression. Thus, whether drug administration is necessary should be evaluated after sufficiently assessing clinical symptoms and risk factors at the time of administration on Day 8 of the initial course.

## Author contributions

**Yuji Mimura:** Conceptualization; data curation; visualization; writing – original draft. **Aya Kobayashi:** Data curation; supervision; visualization; writing – review and editing. **Haruhiko Utazu:** Data curation. **Yuki Matsumoto:** Data curation. **Hiroya Mizusawa:** Conceptualization; supervision; writing – review and editing.

## Conflict of interest

The authors declare no conflict of interest.

## Approval of the research protocol by an Institutional Reviewer Board

This study was approved by the Ethical Institutional Review Board of Shinshu Ueda Medical Center (No. 03‐33).

## Informed consent

Written informed consent was obtained from the patient for publication.

## Registry and the Registration No. of the study/trial

Not applicable.

## References

[1] Charlton OA , Harris V , Phan K , Mewton E , Jackson C , Cooper A . Toxic epidermal necrolysis and Steven–Johnson syndrome: a comprehensive review. Adv. Wound Care 2020; 9: 426–9. 10.1089/wound.2019.0977 PMC7307670 32520664

[2] Sekula P , Dunant A , Mockenhaupt M *et al*. Comprehensive survival analysis of a cohort of patients with Stevens–Johnson syndrome and toxic epidermal necrolysis. J. Invest. Dermatol. 2013; 133: 1197–204. 23389396 10.1038/jid.2012.510

[3] Chang E , Weinstock C , Zhang L *et al*. FDA approval summary: enfortumab vedotin for locally advanced or metastatic urothelial carcinoma. Clin. Cancer Res. 2021; 27: 922–7. 32962979 10.1158/1078-0432.CCR-20-2275

[4] Powles T , Rosenberg JE , Sonpavde GP *et al*. Enfortumab vedotin in previously treated advanced urothelial carcinoma. N. Engl. J. Med. 2021; 384: 1125–35. 33577729 10.1056/NEJMoa2035807 PMC8450892

[5] Rosenberg JE , O'Donnell PH , Balar AV *et al*. Pivotal trial of enfortumab vedotin in urothelial carcinoma after platinum and anit‐programmed death 1/programmed death ligand 1 therapy. J. Clin. Oncol. 2019; 37: 2592–600. 31356140 10.1200/JCO.19.01140 PMC6784850

[6] Nguyen MN , Reyes M , Jones SC . Postmarketing case of enfortumab vedotin‐associated skin reactions reported as Stevens–Johnson syndrome or toxic epidermal necrolysis. JAMA Dermatol. 2021; 157: 1237–9. 34495281 10.1001/jamadermatol.2021.3450 PMC8427493

[7] Francis A , Jimenez A , Sundaresan S , Kelly B . A rare presentation of enfortumab vedtin‐induced toxic epidermal necrolysis. JAAD Case Rep. 2021; 7: 57–9. 33319007 10.1016/j.jdcr.2020.10.020 PMC7727299

[8] Viscuse PV , Marques‐Piubelli ML , Heberton MM *et al*. Enfortumab vedotin for metastatic urothelial carcinoma: a case series on the clinical and histopathologic spectrum of adverse cutaneous reactions from fatal Stevens–Johnson syndrome/toxic epidermal necrolysis to dermal hypersensitivity reaction. Front. Oncol. 2021; 10.3389/fonc.2021.621591. PMC7970171 33747934

[9] Maloney NJ , Ravi V , Cheng K , Bach DQ , Worswick S . Stevens–Johnson syndrome and toxic epidermal necrolysis‐like reactions to checkpoint inhibitors: a systemic review. Int. J. Dermatol. 2020; 59: 183–8. 10.1111/ijd.14811 32052409

